# Investigating the Coating Effect on Charge Transfer Mechanisms in Composite Electrodes for Lithium-Ion Batteries

**DOI:** 10.3390/ijms24119406

**Published:** 2023-05-28

**Authors:** Anna A. Fedorova, Oleg V. Levin, Svetlana N. Eliseeva, Tomaž Katrašnik, Dmitrii V. Anishchenko

**Affiliations:** 1 Faculty of Mechanical Engineering, University of Ljubljana, Aškerčeva 6, SI-1000 Ljubljana, Slovenia; anyfyodorova@gmail.com (A.A.F.); tomaz.katrasnik@fs.uni-lj.si (T.K.); 2 Institute of Chemistry, Saint Petersburg University, 7/9 Universitetskaya nab., St. Petersburg 199034, Russia; svetlana.eliseeva@spbu.ru (S.N.E.); dima_anishenko@mail.ru (D.V.A.)

**Keywords:** conductive binders, intrinsically conductive polymers, coating, composite electrode material, apparent diffusion coefficient, modeling, LIBs

## Abstract

The performance of lithium-ion batteries (LIBs) relies on the characteristics of the cathode material, including both intentionally applied coatings and naturally formed surface layers or binder adhesion. This study investigated the influence of the ion-permeable surface fraction, distribution, and characteristics of the coating on the performance of a lithium iron phosphate (LFP) electrode material. We developed an extended Newman-type half-cell model and examined the impact of coating parameters on the galvanostatic discharge curves of the LFP electrode material. The study found that the ion-permeable surface fraction has a significant influence on the diffusion and charge transfer characteristics of the electrode material. A decrease in the ion-permeable surface fraction leads to a decrease in the measured diffusion coefficients and to an increase in the overall coating resistance of the electrode material. Interestingly, the distribution of the ion-permeable surface also plays a role in the diffusion characteristics, with a coarsely dispersed coating resulting in lower diffusion coefficients. Additionally, the coating characteristics significantly affect the polarization and capacity of the electrode material at different C-rates. The model was used to approximate the experimental discharge curves of the LFP-based composite electrodes with two different compositions, and the simulated data showed satisfactory agreement with the experiment. Thus, we believe that the developed model and its further extension will be useful in numerical simulations that aim to facilitate the search for optimal compositions.

## 1. Introduction

Lithium-ion batteries (LIBs) have become increasingly popular in recent decades due to the widespread use of portable electronic devices such as laptops, mobile phones, and electric scooters, as well as the rise of electric vehicles [1,2,3,4,5,6,7,8,9]. However, the ever-growing demand for power and energy requires the continuous improvement of existing electrode materials.

One way to improve battery power and energy characteristics is to search for and develop new active materials. The work in this field has been conducted for many decades and has led to the availability of a wide variety of materials with higher capacities, energy densities and rate capabilities. At the beginning of such intense research, there were many breakthroughs regarding the discovery of new types of materials. This led to an increase in the gravimetric energy density of LIBs from approximately 90 Wh kg^−1^ at the cell level in the 1990s to the current value of 250 Wh kg^−1^, which has enabled their successful application in electric vehicles, LIB-based grid storages. Today, Li-ion batteries are even being considered for electric planes [10,11]. However, presently, many cheap and easily synthesized materials have been already found. The further development of active materials is costly and leads only to incremental improvements in the electrochemical characteristics LIBs [12].

That is why another way to improve the characteristics of LIBs is becoming increasingly popular, namely, the compositional optimization of the composite material. This approach involves adjusting the electrode material composition by changing the amount and nature of the binder, conductive, and active components. However, keeping the composition evenly distributed all over the electrode is complicated, as it tends to segregate. For example, the aggregation of carbon particles is a common issue that causes uneven electron-conducting additive distribution in composite materials, leading to the formation of isolated particles, contact losses, and capacity reductions [13,14]. Therefore, evenly distributed electron-conducting coatings of active material play an essential role in composite material performance. That is why pre-coating the active material with carbon by annealing various carbon-producing agents is a common approach to improving the electrode material characteristics [15,16,17].

Recent studies have revealed that the coating of composite materials can have complex effects on their performance. It was shown that the uniform distribution of a polydopamine-derived carbon coating reduces the capacity of the active material compared to the inhomogeneous glucose-derived coatings of the same material [18]. It is worth noting that the diffusion coefficients of Li^+^ in the active material appear to be dependent on the coating. The authors believe that such abnormal behavior is related to the hindered intercalation of ions through the coating. However, there is no qualitative and quantitative explanation for this phenomenon, although this would be of great importance for compositional optimization.

In the following article, we want to better understand the interplay between a coating and the charge/discharge characteristics of the electrode material. We achieve this goal by simulating the behavior of composite materials numerically. To the best of our knowledge, mathematical models accounting for the coating-hindered intercalation of ions within composite materials have not yet been developed. Therefore, in the following work, we propose a model that accounts for a composite electrode with various coating patterns and describes the relation between coating and LIB performance.

**Figure 1:**
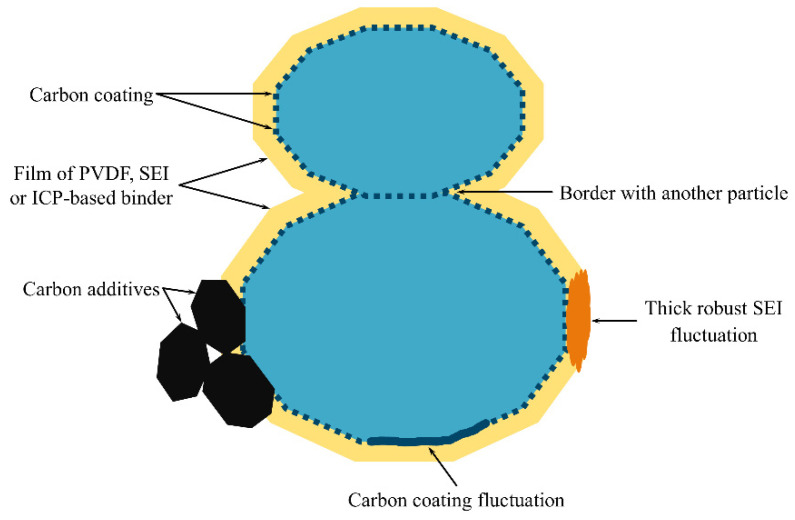
Schematic representation of the factors that inactivate the surface of the electrode material for ion intercalation.

The main assumption of the model is that only a fraction of the active material surface is available for ion intercalation. Figure 1 illustrates several scenarios in which the inactivation of the particle surface occurs, including the formation of inactive spots on the surface due to the aggregation of carbon additives or common boundaries between particles.

First, we investigate the influence of the coating characteristics on the diffusion behavior of a simplified single-particle system with no charge transfer limitations. We show that the presence of inactive spots not only increases the coating resistance, but also deteriorates the diffusion characteristics; the latter is in an agreement with the observations found in [18]. Then, we simulate the influence of the coating on the galvanostatic discharge of the prolonged multi-particle system (coin cell with LFP-coated cathode and lithium anode). The rate capability and the voltage of the coin demonstrates a dramatic dependence on the coating parameters. Then, we compare the galvanostatic discharge curves of the conventional composite to the composite with a conductive binder. Conductive binders are promising additives that are known to improve the electrochemical performance of many active materials [19,20,21]. In the last section, we approximate two sets of experimental data and demonstrate the agreement between the model and the experiment.

## 2. Results and Discussion

### 2.1. Single-Particle Model Accounting for Coating Effects

**Figure 2:**
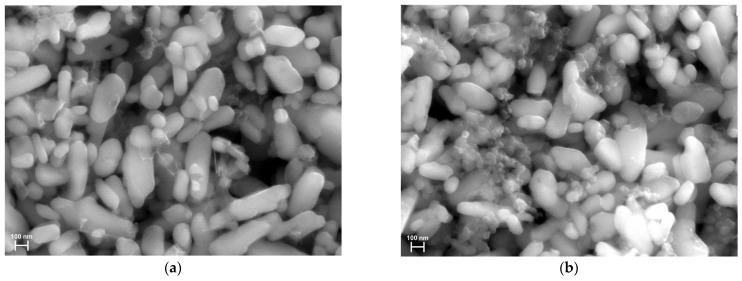
SEM images of LFP electrode. (**a**) Composite with conductive binder. (**b**) Conventional composite.

In this section, we present a single-particle model of the LFP composite material coated with an arbitrary layer, with no charge transfer limitations. Figure 2 shows SEM images of the composite with a conductive binder and the conventional composite. Both samples reveal a noticeable distribution in both the shape and size of the LFP particles. In the case of the composite with carbon black and the conventional, non-conductive binder (Figure 2b), its surface is coated with large agglomerates of carbon black, while in the composite with the conductive binder (Figure 2a), the surface is almost free from carbon agglomerates. The LFP material is known to exhibit anisotropic diffusion, with a higher diffusion coefficient in one direction than in the other two directions. Accounting for the particle shape and size distribution, as well as diffusion anisotropy, would considerably complicate the modeling problem, which is beyond the scope of this work that aims to investigate the coating effects. Thus, we follow the widely accepted approach of using the average particle size and shape, and the isotropic diffusion coefficient of the material [22,23,24,25]. The image shows that a significant number of particles have an elongated shape, resembling a cylinder more than a sphere. Therefore, we adopt the cylindrical geometry of the average particle in our model.

**Figure 3:**
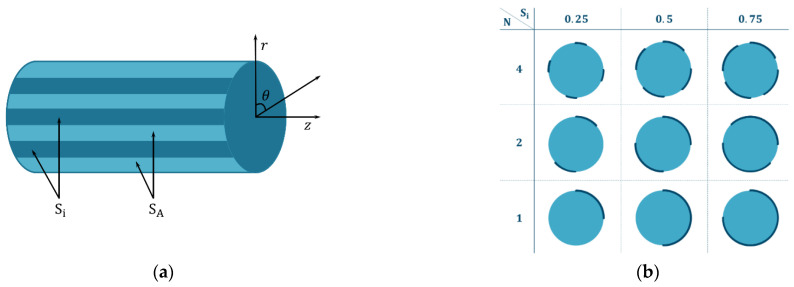
(**a**) A cylindrical particle with a coating described by two parameters, $S_{i}$ and N ($N=8,S_{i}=0.5$), where r is the radial coordinate (distance from the center of the particle); z is the distance along the cylinder; and θ is the angular coordinate. (**b**) Variety of projections of one-particle systems that possess different inactive surface fractions ($S_{i}$) and a different number of inactive spots (N). The thick dark-blue line on the particle border denotes the inactive surface fraction ($\theta \in S_{i}$), while the light-blue color indicates the active surface fraction ($\theta \in S_{A}$).

Let us consider the single-particle system of the LFP electrode material. The particle has the shape of a cylinder and not all parts of the particle surface allow ion intercalation due to the patchy coating. We restrict our consideration to coatings that can be described by two parameters (accounting for all possible coating patterns on a cylinder is an endless task). The first parameter is $S_{i}$, which is the inactive surface fraction that does not allow ion intercalation ($0<S_{i}<1$). From $S_{i}$, one can express the active surface fraction as $S_{A}=1-S_{i}$. The second parameter is the number of inactive spots of equal area and shape N. We consider coating patterns that change only within the angular coordinate θ (nothing changes within the z-axis), Figure 3a. Together with the neglect of the edge effects at the bases of the cylinder, we can transform the three-dimensional cylindrical problem into a two-dimensional radial problem, as shown in Figure 3b.

To develop a single-particle model based on the mentioned assumptions, we analyze the system that consists of one particle of the LFP active material that is immersed in the LiPF_6_ solution (1 mol/L) and has the following parameters. The particle radius $R_{s}=85$ nm. Some parts of the particle surface are capable of fast Li-ion intercalation (negligible charge transfer resistance). Other parts of the particle surface do not intercalate Li-ions. Potential 3.2 V corresponds to the initial concentration of Li-ions in the particle $c_{s,0}$ (1% charge of the LFP material). The potential step from 3.2 V to 3.8 V leads to a decrease in the concentration to $c_{s,99\%}$ (99% charge of the LFP material). The lithium diffusion coefficient in the solid for this system is $D_{s}$. Details of the single-particle potential step simulation can be found in Appendix A.

The simulated potential step from 3.2 V to 3.8 V results in the current vs. time dependence (current transient). This dependence enables the apparent diffusion coefficient $D_{App}$ to be calculated if one knows the particle surface area $A_{particle}$ and concentration change corresponding to a given potential step $∆c_{s}=c_{s,0}-c_{s,99\%}$. Calculations of $D_{App}$ can be performed using the Cottrell relationship [26,27,28,29,30]:

(1) $$I\left(t\right)=\frac{FA_{particle}\sqrt{D_{App}}∆c_{s}}{\sqrt{\pi t}},$$

where $D_{App}$ is the apparent diffusion coefficient, $I\left(t\right)$ is the current, and F is the Faraday’s constant. $D_{App}$ measured by the Cottrell relationship characterizes the diffusion at the beginning of the charging process. Another parameter that can be used as a diffusion characteristic is the time $t_{90\%}$, which tells how long it takes to add 90% of $∆c_{s}$.

We apply the potential step to a simulated idealized system with a negligible charge transfer resistance and a diffusion coefficient with a constant value for the lithium in the lithium iron phosphate $D_{s}$. Hence, one large potential step can be used instead of the usual approach, in which lots of small potential steps are applied.

**Figure 4:**
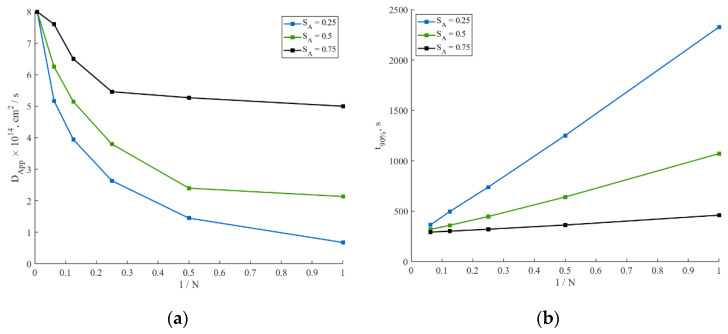
Dependence of the diffusion characteristics of the simulated system on the coating parameters. (**a**) Dependence of the apparent diffusion coefficient $D_{App}$ on the reciprocal number of inactive spots N at three different values of active surface fraction $S_{A}$. (**b**) Dependence of the diffusion time $t_{90\%}$ on the reciprocal number of inactive spots N at three different values of active surface fraction $S_{A}$.

Dependences of $D_{App}$ and $t_{90\%}$ on particle coatings are represented in Figure 4a,b, respectively.

**Figure 5:**
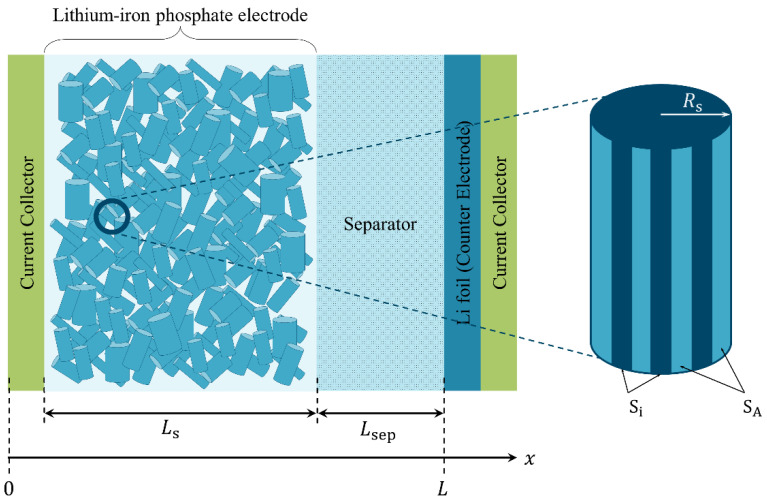
Schematic representation of the cross section of cell configuration.

It is clear from Figure 4 that in the case of the finely dispersed coating (1/N→0), the value of the apparent diffusion coefficient $D_{App}$ remains close to the value of the Li^+^ diffusion coefficient in the lithium iron phosphate $D_{s}=8\times 10^{-14}$ cm^2^/s. However, this is not the case for the coarsely dispersed coating (1/N→1). A closer look at Figure 5 reveals that at high values of $S_{A}$, the $D_{App}$ value does not differ much from $D_{s}$. Thus, when $S_{A}$ equals 0.75 (75% of surface allows ion intercalation), a change in the particle coating from finely dispersed to coarsely dispersed leads to a decrease in the $D_{App}$ value by ~38%. However, if $S_{A}$ equals 0.25 (25% of surface allows ion intercalation), there is an order of magnitude decrease in the value of $D_{App}$ when the coating changes from finely dispersed to coarsely dispersed. Another interesting observation from the graph is that, in some cases, a finer coating with a smaller $S_{A}$ may give better diffusion characteristics than a coarser coating with a bigger $S_{A}$. For example, this is clearly seen for a fine coating (1/N=0.0625) with $S_{A}=0.25$ and a coarse coating (1/N=1) with $S_{A}=0.5$.

Figure 4b demonstrates the dependence of the diffusion time *t*_90%_ on the reciprocal number of inactive spots 1/N for three different values of the active surface fraction $S_{A}=\{0.25,0.5,0.75\}$. One can infer that the time $t_{90\%}$ has a nearly linear dependence on the reciprocal number of inactive spots 1/N. Additionally, the active surface fraction $S_{A}$ affects the angle of $t_{90\%}$ vs. 1/N dependence. If 75% of the particle surface allows ion intercalation ($S_{A}=0.75$, black line in Figure 4b), no significant change in the diffusion time is observed ($t_{90\%}$ increases ~1.6 times when the coating changes from finely dispersed to coarsely dispersed). However, if only 25% of the particle surface allows ion intercalation ($S_{A}=0.25$, blue line in Figure 4b), a significant change in the diffusion time is observed ($t_{90\%}$ increases almost one order of magnitude when the coating changes from finely dispersed to coarsely dispersed). Hence, the active surface fraction $S_{A}$ strongly affects the angle of the $t_{90\%}$ vs. 1/N dependence; the lower the $S_{A}$ value, the more dramatic the deterioration of the diffusion characteristics caused by the coarsely dispersed coating is.

By now, we have demonstrated that the finely dispersed coating has an insignificant influence on the diffusion characteristics of the system. However, such a coating still plays a role in the surface resistance of the particle coating $R_{COAT}$. If the particle has an inactive surface fraction $S_{i}$ with the surface resistance $R_{\infty }=\infty$ Ohm·m^2^ and an active surface fraction $S_{A}$ (covered with the film) with the surface resistance $R_{film}$ Ohm·m^2^, we can apply the summation rule for parallel resistances:

(2) $$\frac{1}{R_{COAT}}=\frac{S_{i}}{R_{\infty }}+\frac{S_{A}}{R_{film}}.$$

Thus, we get the following expression for *R*_COAT_:

(3) $$R_{COAT}=\frac{R_{film}}{S_{A}}.$$

Therefore, despite a slight effect on the diffusion characteristics, a finely dispersed coating affects the polarization of the system, so, a finely dispersed coating can be taken into account via the resistance $R_{COAT}$.

In this section, we demonstrated the impact of a coating on the measured diffusion characteristics for the case of a single-particle system with no charge transfer and ohmic limitations. We found that a finely dispersed coating leads to a slight dependence of diffusion characteristics on the active surface fraction $S_{A}$, as shown in Figure 4. However, a coarsely dispersed coating results in a significant deterioration of the diffusion characteristics with a decrease in $S_{A}$. In real systems, the situation is more complex: some parts of the coating may have a little effect on the diffusion characteristics due to a small average inactive spot size, while other parts may have large inactive surface spots due to fluctuations in the coating fabrication, electrochemical restructuring, etc. (Figure 1). The parts of the particle surface with a finely dispersed coating can be considered as an active surface by adjusting the value of $R_{COAT}$; then, the parts covered with large inactive spots should be considered as an inactive surface. This assumption simplifies the problem of the simultaneous presence of big and small inactive spots to the problem of big inactive spots.

### 2.2. General Model Accounting for Coating Effects

The single-particle model does not take into account the distribution of the potential and the electrolyte distribution in the porous composite electrode. Thus, the development of a full-scale model is more preferable for precise numerical simulations of prolonged multi-particle systems.

In this section, we utilize the widely known Newman approach in order to model the electrochemical behavior of the cell. This approach involves a set of five equations that describe the kinetics and dynamics of the cell, including the concentration of lithium in both the solid and electrolyte phases, as well as the potential distribution in these phases. These equations are bounded by the Butler–Volmer equation, and they provide us with a comprehensive understanding of the internal behavior of the cell [22]. However, if we want to model the impact of different surface coatings on the electrode particles, we need to extend the Newman model. Specifically, we must add an additional dimension in order to account for the presence of active and inactive surface fractions ($S_{A}$ and $S_{i}$, respectively). The concentration profile inside a particle with a patchy coating will depend on the angular coordinate θ, which must be included in the model. Therefore, we modify the Newman model by adding the angular coordinate. A schematic representation of the cross-section of the cell modeled and described in this study is shown in Figure 5.

In this study, we investigate the electrochemical performance of a cell containing a positive LiFePO_4_ electrode, a negative Li metal electrode and a porous separator. Several coatings of the positive electrode were considered. Although in commercial lithium-ion batteries the negative electrode is typically composed of an intercalation material like the positive electrode, in this article, we consider the negative electrode to be an ideal lithium metal in order to focus mainly on the electrochemical performance of the positive LiFePO_4_ electrode with different coatings.

Here, we expand the widespread Newman’s approach [22,23,24,25] in order to model and describe the internal electrochemical behavior of the cell, whose cross section is presented in Figure 5. In general, the equations will be the same; however, an additional dimension is needed to characterize the size and distribution of the ion-impermeable spots. We used the cylindrical geometry of the particles (neglecting the edge effects at the bases of cylinders) for two reasons. (1) The composite material under consideration has prolonged particles, that look like cylinders. (2) Using an extension of the Newman model to account for the sphere surface coating would demand two additional dimensions, which would excessively complicate the computational problem.

Next, we describe in detail the equations used to model the behavior of the cell. We denote the thickness of the cell by x (distance from the current collector to the ideally reversible lithium counter electrode). Thus, x=0 is the beginning of the cell and $x=L=L_{s}+L_{SEP}$ is the overall thickness of the cell, where $L_{s}$ and $L_{SEP}$ are the thicknesses of the solid (electrode) and separator, respectively (see Figure 5).

#### 2.2.1. Mass Conservation in the Solid

The lithium concentration changes in the solid particles in accordance with the diffusion equation for cylindrical coordinates:

(4) $$\frac{\partial c_{s}(r,\theta ,x,t)}{\partial t}=\frac{1}{r}\frac{\partial }{\partial r}\left(D_{s}r\frac{\partial c_{s}(r,\theta ,x,t)}{\partial r}\right)+D_{s}\frac{1}{r^{2}}\frac{\partial^{2}c_{s}(r,\theta ,x,t)}{\partial \theta^{2}},$$

where $c_{s}(r,\theta ,x,t)$ is the concentration of lithium in the solid phase depending on the radial coordinate r, angular coordinate *θ*, distance across the cell *x* and time t. $D_{s}$ is the diffusion coefficient of lithium in the solid.

The initial concentration of lithium in the solid is equal to the following:

(5) $$c_{s}\left(r,\theta ,x,0\right)=c_{s,0},$$

where $c_{s,0}$ is the lithium concentration in the solid at the beginning of the simulation.

The boundary conditions are given by the following equation:

(6) $$\left\{\begin{array}{l} {\left.D_{s}\frac{\partial c_{s}\left(r,\theta ,x,t\right)}{\partial r}\right|}_{r=R_{s}}=-j\left(\theta ,x,t\right),\theta \in S_{A}\\ {\left.D_{s}\frac{\partial c_{s}\left(r,\theta ,x,t\right)}{\partial r}\right|}_{r=R_{s}}=0,\theta \notin S_{A} \end{array},\right.$$

where $j\left(\theta ,x,t\right)$ is the lithium flux across the active surface fraction *S_A_*.

#### 2.2.2. Charge Conservation in the Solid

The potential distribution in the electrode is represented by the following equation:

(7) $$\frac{\partial }{\partial x}\left(\sigma^{eff}\frac{\partial }{\partial x}\varphi_{s}\left(x,t\right)\right)=a_{s}Fj\left(x,t\right),$$

where $\varphi_{s}\left(x,t\right)$ is the potential in the solid that depends on the distance across the cell *x* and time t, $\sigma^{eff}=\sigma \varepsilon_{s}^{brug}$ is the effective solid conductivity, $\varepsilon_{s}$ is the volume fraction of the solid phase, brug is the Bruggeman’s coefficient, and *F* is Faraday’s constant. $a_{s}$ is the specific surface area of the porous electrode material, and j(x,t) is the *θ*-averaged lithium flux through the surface of a particle located at *x* position in the electrode coordinate, at the time *t*.

The current that flows through the electrode is bounded by the following conditions:

(8) $$-\sigma^{eff}\frac{\partial \varphi_{s}\left(0,t\right)}{\partial x}=\sigma^{eff}\frac{\partial \varphi_{s}\left(L,t\right)}{\partial x}=\frac{I}{A},$$

where *I* is the applied current density, and *A* is the geometric surface area of the electrode.

#### 2.2.3. Mass Conservation in the Electrolyte

A material balance equation in the electrolyte is represented in the following form:

(9) $$\frac{\partial }{\partial t}\left(\varepsilon_{e}c_{e}\left(x,t\right)\right)=\frac{\partial }{\partial x}\left(D_{e}^{eff}\frac{\partial }{\partial x}c_{e}\left(x,t\right)\right)+\left(1-t_{+}^{0}\right)a_{s}j\left(x,t\right),$$

where $c_{e}\left(x,t\right)$ is the concentration of lithium in the electrolyte, $D_{e}^{eff}=D_{e}\varepsilon_{e}^{brug}$ is the effective electrolyte diffusivity, $\varepsilon_{e}$ is the volume fraction of the electrolyte, $D_{e}$ is the diffusion coefficient of lithium in the electrolyte, and $t_{+}^{0}$ is the Li^+^ transference number.

The initial concentration of lithium in the electrolyte is determined as follows:

(10) $$c_{e}\left(x,0\right)=c_{e,0},$$

where $c_{e,0}$ is the lithium concentration in the electrolyte at the beginning of the simulation.

For the cell under consideration, the ion deposition/dissolution at the ideal lithium metal is represented as follows:

(11) $$\frac{\partial c_{e}(L,t)}{\partial x}=I\frac{1-t_{+}^{0}}{D_{e}^{eff}F},$$

#### 2.2.4. Charge Conservation in the Electrolyte

Charge conservation equation in the electrolyte phase is formulated in Equation (12):

(12) $$-\kappa^{eff}\frac{\partial }{\partial x}\varphi_{e}\left(x,t\right)+\kappa^{eff}\frac{RT}{F}\left(1+\frac{\partial \ln f_{A}}{\partial \ln c_{e}\left(x,t\right)}\right)\left(1-t_{+}^{0}\right)\frac{\partial }{\partial x}\ln c_{e}\left(x,t\right)=a_{s}Fj\left(x,t\right),$$

where $\varphi_{e}\left(x,t\right)$ is the electrolyte potential, $\kappa^{eff}=\kappa_{e}\varepsilon_{e}^{brug}$ is the effective electrolyte conductivity, $\kappa_{e}$ is the electrolyte conductivity, R is the universal gas constant, T is the cell temperature, and $f_{A}$ is the activity coefficient of the electrolyte.

The corresponding boundary conditions are as follows:

(13) $$\frac{\partial \varphi_{e}(0,t)}{\partial x}=\frac{\partial \varphi_{e}(L,t)}{\partial x}=0.$$

#### 2.2.5. Butler–Volmer Equation

The rate of lithium movement between the solid and electrolyte phases is modeled via the Butler–Volmer equation:

(14) $$\left\{\begin{array}{l} j\left(\theta ,x,t\right)=kc_{e}^{1-\alpha }{\left(c_{s,max}-c_{s,e}\right)}^{1-\alpha }c_{s,e}^{\alpha }\cdot \left\{exp\left(\frac{\left(1-\alpha \right)F}{RT}\eta \left(x,t\right)\right)-exp\left(-\frac{\alpha F}{RT}\eta \left(x,t\right)\right)\right\},\theta \in S_{A}\\ j\left(\theta ,x,t\right)=0,\;\;\theta \notin S_{A} \end{array}\right.$$

where *k* is the reaction rate constant, α is the charge transfer coefficient, $c_{s,max}$ is the maximum concentration of lithium in the solid, and $c_{s,e}$ is the surface concentration of lithium in a cylindrical electrode particle and the overpotential that is $\eta \left(\theta ,x,t\right)$ equal to the following:

(15) $$\eta \left(\theta ,x,t\right)=\varphi_{s}\left(x,t\right)-\varphi_{e}\left(x,t\right)-U_{OCP}\left(\frac{c_{s}}{c_{s,max}}\right)-j\left(\theta ,x,t\right)FR_{film},$$

where $U_{OCP}$ is the open-circuit potential of the electrode, and $R_{film}$ is the film resistance.

### 2.3. Study of Coating Effects on Charge/Discharge Characteristics of LFP Material

In this section, we apply the developed model to study the influence of coating parameters on the galvanostatic discharge curves of the LFP material at different C-rates. The parameters used for simulation can be found in Table 1. The dependence of the $U_{OCP}$ curve of the positive LFP electrode on the lithium solid concentration was taken from reference [25].

**Figure 6:**
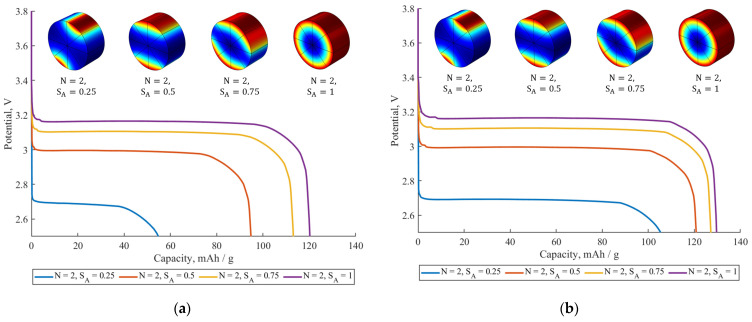
The influence of the active surface fraction *S_A_* on galvanostatic discharge curves at 0.5C for two materials with $R_{film}=4.15$ Ωm^2^ for both materials and diffusion coefficients: (**a**) $D_{s}=1.33\cdot 10^{-14}$cm^2^/s, (**b**) $D_{s}=4\cdot 10^{-14}$ cm^2^/s. Inset: colors are expressing Li^+^ concentration in the particle–blue for lower concentrations, red for higher ones.

Figure 6 compares the effect of the active surface fraction $S_{A}$ on the galvanostatic discharge curves at 0.5C for two different materials with different diffusion coefficients for the constant value of the parameter N. Figure 6a corresponds to the material with a relatively slow diffusion coefficient ($D_{s}=1.33\times 10^{-14}$ cm^2^/s). As one can see, the capacity decreases from 121 mAh when $S_{A}=1$ to less than 60 mAh when $S_{A}=0.25$. Thus, a high fraction of inactive surface leads to a significant deterioration in the material discharge capacity. This can be interpreted in terms of a change in the diffusion length and diffusion geometry (see the insets in Figure 6a). The more inactive surface fraction the particle has, the greater the distance in a wider range of directions the Li^+^ ions must overcome in order to fill the particle. Therefore, the “real” diffusion geometry is no longer cylindrical. For example, the diffusion inside the particle with the coating characteristics of $N=2\;\mathrm{and}\;S_{A}=0.5$ is more like 1D planar diffusion rather than 2D cylindrical diffusion. This leads to an increase in the diffusion time and, consequently, to a capacity reduction at a given discharge rate. Another effect that can be noticed from Figure 6a is a significant drop in the discharge voltage plateau with a decrease in the active surface fraction $S_{A}$. This happens because a lower active surface fraction leads to a greater coating resistance $R_{COAT}$ in accordance with Equation (3). Therefore, the active surface fraction has a significant influence on both the voltage and capacity of the considered system.

Figure 6b corresponds to the material with a diffusion coefficient of $D_{s}=4\cdot 10^{-14}$ cm^2^/s. It is clear from the graph that the influence of $S_{A}$ on the discharge capacity decreases at a higher diffusion coefficient value. The capacity reduces from 130 mAh to 105 mAh when *S_A_* changes from 1 to 0.25. Concerning the discharge voltage of the samples presented in Figure 6a,b, it remains nearly the same. This happens because the value of the $R_{film}$ resistance remains constant for the systems considered in Figure 6.

**Figure 7:**
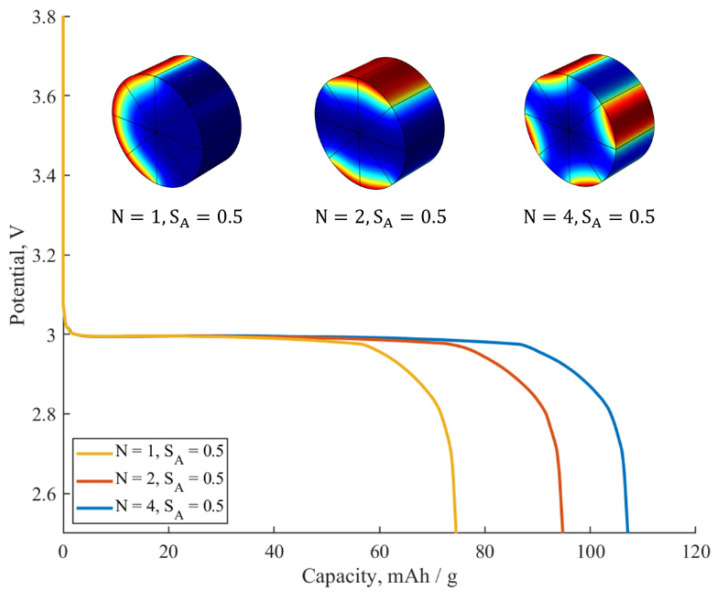
The influence of the number of ion-impermeable surface spots N on the galvanostatic discharge curves at 0.5C. $S_{A}=0.5$, $R_{film}=4.15$ Ωm^2^. Inset: colors are expressing Li^+^ concentration in the particle–blue for lower concentrations, red for higher ones.

Now, we turn to the study of the coating distribution on the performance of the electrode material at a given C-rate (0.5C). Figure 7 compares three samples that differ from each other only by the number of inactive spots N. The capacity of the sample with the highest N value (N=4) reaches 108 mAh/g, while the discharge capacity of the sample with the lowest N value (N=1) is about 75 mAh/g. Therefore, at moderate values of active surface fraction ($S_{A}=0.5$), a significant deterioration in the diffusion characteristics is observed with the decrease in the number of inactive spots N. This agrees with the inference made in the first section of this work. Thus, the agglomeration of additives into big lumps that inactivate the electrode surface leads to a decrease in the value of the discharge capacity at a given C-rate.

Concerning the voltage plateau, it remains the same for all samples in Figure 7, as it does not depend on N and depends only on $S_{A}$ and $R_{film}$. Therefore, the coating distribution influences predominantly the discharge capacity of the electrode material, while having a negligible influence on the voltage characteristics.

**Figure 8:**
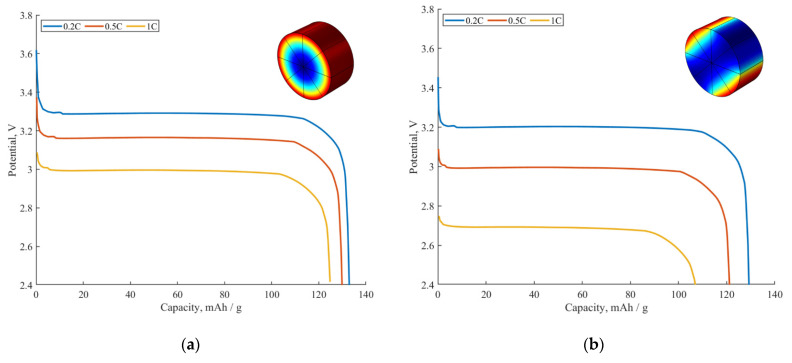
The influence of active surface fraction $S_{A}$ on galvanostatic discharge curves at different C-rates (0.2C, 0.5C, 1C), $R_{film}=4.15$ Ωm^2^. (**a**) Completely active surface ($S_{A}=1$). (**b**) Half-inactivated surface ($S_{A}=0.5$). Inset: colors are expressing Li^+^ concentration in the particle–blue for lower concentrations, red for higher ones.

Figure 8 demonstrates the influence of the active surface fraction $S_{A}$ on the simulated discharge curves at different C-rates. One can see that the sample with a half-inactivated surface (Figure 8b) has a higher voltage drop at any given C-rate in comparison to the sample with a completely active surface (Figure 8a). For instance, at the 1C discharge rate, we observe a significant decrease in the discharge voltage plateau down to 2.7 V for the sample with the half-inactivated surface ($S_{A}=0.5$), while the sample with a completely active surface ($S_{A}=1$) demonstrates a smaller decrease in the voltage plateau down to ~3 V.

Regarding the rate capability, it is higher for the sample with a higher active surface fraction. For example, at the 1C discharge rate, the sample presented in Figure 8a gives a ~124 mAh/g capacity (90% of the theoretical maximum capacity of 138 mAh/g), while the sample presented in Figure 8b gives a capacity of 107 mAh/g at the 1C discharge rate (77% of the theoretical maximum capacity value of 138 mAh/g). Therefore, a high active surface fraction is of a great importance for both the better rate capability and lower polarization of the electrode material.

**Figure 9:**
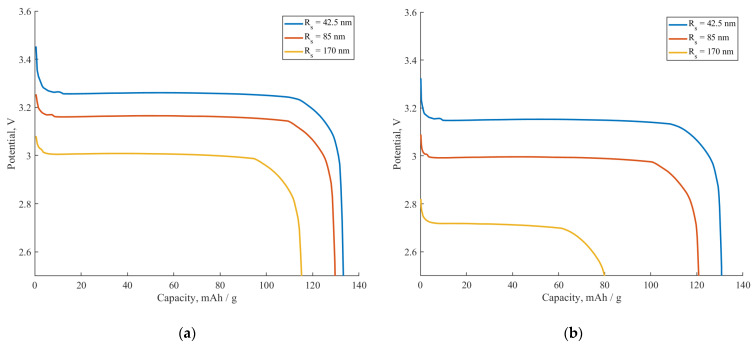
The influence of particle size $R_{s}$ on galvanostatic discharge curves at 0.5 C-rate, $R_{film}=4.15$ Ωm^2^. (**a**) Completely active surface ($S_{A}=1$). (**b**) Half-inactivated surface ($S_{A}=0.5$).

It is a well-known fact that the size of active material particles influences the rate capability and voltage of the cell. For that reason, we investigated the interconnection between the particle size and discharge characteristics at different active surface fractions. Figure 9 demonstrates the influence of the particle radius $R_{s}$ on the galvanostatic discharge curves simulated at a 0.5C current. Figure 9a ($S_{A}=1$) represents the case of the completely active surface, while Figure 9b corresponds to the half-active surface ($S_{A}=0.5$). As can be seen, an increase in the particle radius leads to poorer voltage and capacity characteristics in both cases. However, the deterioration of the cell characteristics is much more profound in the case of the composite with a half-active surface. Thus, bigger particles have greater portions of their bulk that are poorly accessible by non-permeable coatings, which in turn have a greater effect on the capacity and C-rate dependencies.

**Figure 10:**
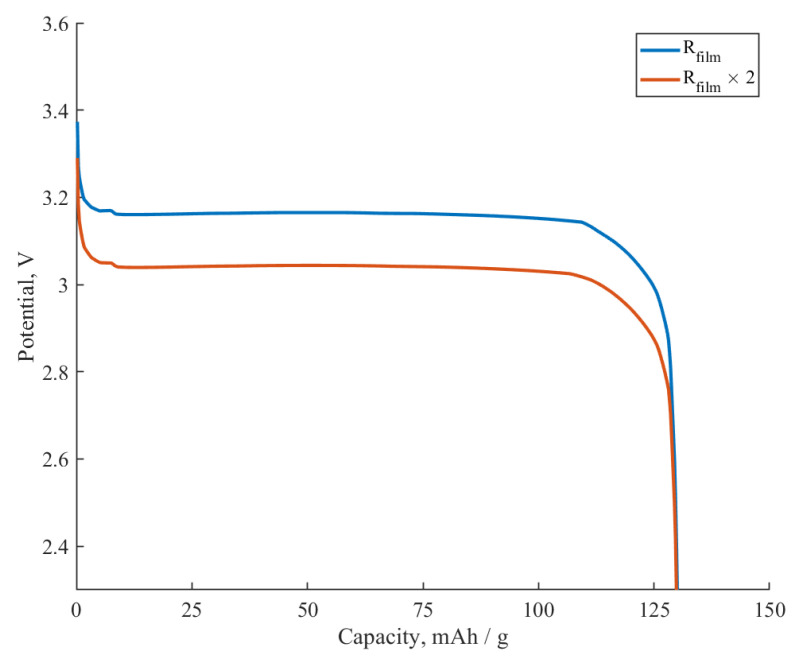
The influence of the film resistance $R_{film}$ on the galvanostatic discharge curve at a 0.5C rate ($S_{A}=1$).

The resistance of the film $R_{film}=4.15$ Ωm^2^ covering the active surface of the lithium iron phosphate material has a predictable influence on the discharge potential, as shown in Figure 10. Thus, a two-fold increase in the $R_{film}\times 2=8.3$ Ωm^2^ leads to a decrease in the discharge voltage plateau of the given electrode material from 3.16 V to 3.04 V. We should note here that, in accordance with Equation (3), for the completely active surface ($S_{A}=1$), the film resistance is equal to the coating resistance$:R_{film}=R_{COAT}$. A change in the $R_{film}$ value can be caused by many factors, including, but not limited to, the following: SEI formation (influenced by electrolyte composition, impurities, and coating), coating fabrication conditions, and amount and nature of the additives (conventional additives, conductive polymeric additives). Therefore, we obtained the expected result: the resistance of the film covering the active material affects the polarization of the electrode materials and does not affect the capacity.

In this section, we showed how coating parameters affect the discharge capacity and polarization of the electrode material. The active surface fraction ($S_{A}$) predominantly influences the polarization of the electrode with a finely dispersed coating (1/N→0), while with a coarsely dispersed coating, the $S_{A}$ has a complex effect, influencing both the capacity and the polarization of the electrode. Concerning the number of inactive spots (N), it affects the discharge capacity and does not affect the polarization of the electrode. The developed model allows one to calculate the dependencies of the discharge capacity and voltage on the coating characteristics for an arbitrary electrode material. Additionally, the approximation of the experimental data with the model enables the values of the kinetic parameters and coating characteristics to be extracted.

### 2.4. Approximation of Experimental Galvanostatic Discharge Curves Recorded at Different C-Rates with the Developed Model

**Figure 11:**
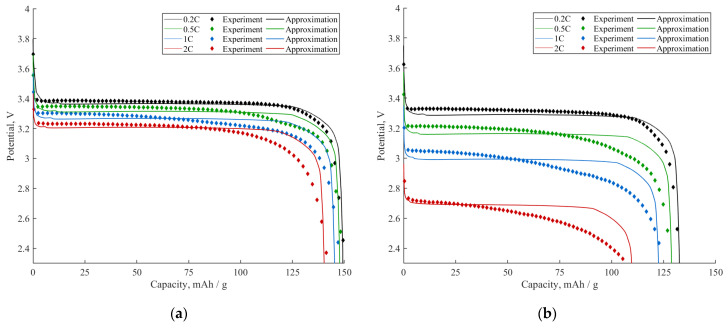
Approximation of experimental galvanostatic discharge curves recorded at different C-rates (0.2C, 0.5C, 1C, 2C) by the developed model. (**a**) Composite electrode with a conductive binder. (**b**) Conventional composite electrode. The values of parameters as in Table 1.

In this section, we approximate two sets of experimental data with the developed model. The first set of data contains the discharge curves of the conventional LFP_coated_ + C + PVDF composite material at different C-rates (0.2C, 0.5C, 1C, 2C). The second set contains the discharge curves of the composite with the addition of the PEDOT:PSS-CMC conductive binder and a reduced amount of PVDF and C additives. The conventional composite has the following composition: 84 wt% LFP_coated_; 8 wt% C; 8 wt% PVDF. Additionally, the LFP_coated_ particles are pre-coated and, thus, 3.6% of their mass is carbon coating. Such a composition has a theoretical capacity of ~138 mAh/g. The composite with the conductive binder has the following characteristics: 92 wt% LFP_coated_; 2 wt% PEDOT:PSS; 2 wt% CMC; 2 wt% C; 2 wt% PVDF. The particles of LFP_coated_ are the same as those in the conventional composite; thus, 3.6% of their mass is also carbon coating. The theoretical gravimetric capacity of such a composite is ~151 mAh/g. The discharge curves of both composites and their approximations by the developed model are illustrated in Figure 11.

One can see from Figure 11 that the compared samples differ both in rate capability and polarization. The sample with the conductive binder shows a high rate capability, maintaining approximately 93% of its theoretical capacity (~151 mAh/g) at a 2C discharge rate (Figure 11a). On the other hand, the conventional composite retains only 77% of its theoretical capacity (~138 mAh/g) at a 2C discharge rate (Figure 11b). Hence, it can be concluded that a conventional composite has a lower active surface fraction $S_{A}$ or/and more coarsely dispersed coating (a lower number of inactive spots N). This is probably due to the higher fraction of ion-impermeable additives in the conventional composite compared to the composite with the conductive binder, as can be seen in the SEM images (Figure 2). One more reason for the reduced value of $S_{A}$ may be the formation of dense SEI layers on parts of the LFP surface in the case of the conventional composite, and the prevention of the formation of dense SEI layers in case of the composite with the conductive binder. The conventional composite has a more coarsely dispersed coating, as carbon additives are known to form agglomerates during electrochemical cycling. A reduced active surface fraction $S_{A}$ and coarsely dispersed coating result in a deterioration in the diffusion characteristics and, consequently, in a reduced discharge capacity at high C-rates.

Concerning a drop in the discharge voltage, it is much more profound for the cell with a conventional composite electrode. Thus, increasing the discharge rate from 0.2C to 2C leads to a decrease in the discharge voltage by more than 600 mV for the cell with a conventional composite electrode; meanwhile, for the composite electrode with a conductive binder, the same change in discharge C-rates results only in a ~160 mV decrease in the voltage. The latter implies that the addition of a conductive binder and a reduction in the amount of carbon and PVDF additives in the composite electrode lead to a significant decrease in the coating resistance $R_{COAT}$. This effect occurs for several reasons. First, as mentioned above, the conventional composite electrode has a lower active surface fraction, which leads to the increase in the coating resistance $R_{COAT}$, in accordance with Equation (3). Second, the resistance of the conductive binder film $R_{film}$ is likely to be lower than the resistance of the PVDF+SEI film covering the conventional composite electrode particles, which influences the coating resistance via Equation (3). Both these factors result in the significant decrease in the discharge voltage of the cell with a conventional composite electrode. Thus, the addition of a conductive binder enables the coating resistance to be reduced by several times. A comparison of the coating resistances measured via Electrochemical Impedance Spectroscopy (EIS) for both the conventional composite and composite with a conductive binder can be found in Appendix B.

One may argue that solid conductivity is another factor that can influence the discharge voltage plateau of the cell. However, the data in the literature on the effective solid conductivity for such types of electrodes (LFP, different coatings, compositions and sizes) varies in the range of 5 × 10^−4^–5 S/m. Thus, such values of effective solid conductivity result in a maximum of a 30 mV shift in the voltage plateau (Appendix C) and lead to no change in the discharge capacity.

An approximation of the two sets of experimental galvanostatic discharge curves (Figure 11) was performed using the model described in Section 2. The approximation steps were as follows. At first, we noted that the composite electrode with a conductive binder demonstrated a good rate capability and low polarization values. This implies that the composite electrode had a high value of active surface fraction $S_{A}$. A high value of $S_{A}$ ($S_{A}>0.75$) allows one to neglect the impact of the coating on the diffusion characteristics of the electrode (see Figure 4). In this case, the coating had an influence on the charge transfer characteristics only and, thereby, could be accounted for by parameter $R_{COAT}$. Such an approach allowed us to estimate the value of the diffusion coefficient of lithium in the solid (*D*_s_) by approximating the first set of experimental discharge curves (composite electrode with conductive binder) with the model; see Figure 11a. The obtained value ($D_{s}=4\cdot 10^{-14}$cm^2^/s) was in accordance with the values used in the works [23,31]. Then, we used the same value of $D_{s}$ to approximate the set of discharge curves of the conventional composite electrode (because $D_{s}$ is a bulk characteristic of the material and does not depend on the coating and additives). The latter approximation allowed us to obtain values of the coating characteristics ($S_{A},N,R_{COAT}$) of the conventional composite electrode. The values of the other parameters used in the simulation are presented in Table 1.

As one can see, the model satisfactorily describes the experimental curves. The discrepancies that are observed closer to the end of the discharge process are most certainly related to the distribution of the particle shapes and sizes as it was shown in the work [23]. According to the above approximations, a four-fold reduction in the carbon and PVDF additives (from 8% to 2%) and the simultaneous use of a conductive binder leads to two-fold increase in the active surface fraction of the active material and to a ten-fold decrease in the coating resistance $R_{COAT}$ of the LFP material.

## 3. Materials and Methods

All reagents were used without further purification. PEDOT:PSS (1.3 wt% aqueous Dispersion), ethylene carbonate (EC), dimethyl carbonate (DMC), LiPF_6_, polyvinylidene fluoride (PVDF) and N-methylpyrrolidone (NMP) were purchased from Sigma-Aldrich (Burlington, MA, United States.). Carbon-coated LiFePO_4_ (LFP) was purchased from Phostech Lithium Inc. (Montreal, QC, Canada). Carboxymethylcellulose (CMC) was purchased from MTI Corp. (Richmond, CA, USA). Conductive carbon black “Super P” was purchased from Timcal Inc. (Bodio, Switzerland).

The composite with a conductive binder was prepared via the mechanical mixing of LFP, PEDOT/PSS/CMC 1:1 aqueous dispersion and carbon black in the following ratio: 94 wt% LFP; 2 wt% PEDOT:PSS; 2 wt% CMC; 2 wt% C; 2 wt% PVDF. The other composite was of a conventional C+PVDF+LFP composition (84 wt% LFP; 8 wt% C; 8 wt% PVDF).

The doctor blade was used to cast the resulting slurry uniformly onto aluminum foil, which served as a current collector. The slurry was dried at 80 °C under vacuum and roll-pressed. The characterization of the morphology of the prepared composites was performed via scanning electron microscopy SUPRA 40VP (Carl Zeiss, Berlin, Germany). Electrochemical measurements were conducted in standard two-electrode coin-type half cells (CR2032) with a lithium anode. The cells were assembled in an argon-filled glove box (Unilab, Fort Lauderdale, FL, USA) using a Celgard 2325 membrane as separator and 1 mol/L of LiPF_6_ in a 1:1 mixture of EC/DMC as an electrolyte. The electrochemical performance tests were carried out on an automatic galvanostatic charge–discharge battery cell test instrument (Neware Co., Hong Kong, China) in a potential range between 2.2 and 4.0 V (vs. Li/Li^+^) at 25 °C. All capacity values were normalized by the total weight of the cathode excluding the current collector.

The physics-based model of the LFP cell was developed in the Multiphysics (MP) software (COMSOL Inc., Palo Alto, CA, USA). The model was based on the well-known Newman approach [22,23,24,25], additionally accounting for the non-uniform coating of LFP particles.

## 4. Conclusions

In this study, we investigated the impact of coating characteristics and patterns on the performance of cathode materials. We first focused on the diffusion characteristics of a coated composite material, and developed a single-particle model to analyze the effects of the inactive surface fraction of the coated particle on the apparent diffusion coefficient. Our results demonstrated that an increased amount of inactive surface area led to a decrease in the apparent diffusion coefficient, as measured by the Cottrell approach. Interestingly, the distribution of inactive surface spots also had a significant influence on the diffusion characteristics. A finely dispersed coating led to a relatively weak deterioration of diffusion characteristics with an increase in the inactive surface fraction, while a coarsely dispersed coating led to a sharp reduction in the measured diffusion coefficients with an increase in the inactive surface fraction.

To investigate the influence of the non-uniform surface coating on the electrochemical properties of the cell, we used an extended Newman-type model. The model included a separator, reversible lithium anode and a porous composite electrode material with additional parameters accounting for the coating of the LFP particles. The simulated galvanostatic discharge curves of the material were significantly affected by the coating characteristics. Thus, the finely dispersed coating predominantly influenced the observed voltage drop in the discharge curves, while the coarsely dispersed coating influenced both the voltage drop and the rate capability in the electrode material at different C-rates. The simulated discharge curves of the samples with the coarsely dispersed coating demonstrated a faster capacity fade, starting at lower C-rates in comparison to the finely dispersed samples.

To verify our developed model, we performed experimental investigations on two LFP-based composite materials. Our results showed that the conventional composite had a lower rate capability compared to the composite with the conductive binder. This indicated that the higher amount of additives in the conventional composite led to the excessive blocking of the LFP surface, which resulted in the deterioration of the diffusion characteristics. In contrast, the conductive binder prevented the formation of robust SEI layers on the surface of the particles, which enlarged the Li^+^ insertion cross-section area, accelerating ion intercalation. Additionally, the ICP-based binder enabled very fast ion transport within itself, reducing the coating resistance. Our model successfully approximated the experimental curves, indicating its usefulness in numerical experiments aimed at finding the optimal coating composition for a given composite. Overall, our findings suggest that conductive binders have potential applications in solving LIB performance problems.

## Figures and Tables

**Table 1:** Parameters used in the simulation.

Symbol	Units	Positive Electrode, Conventional Composite	Positive Electrode with Conductive Binder	Separator
α	unitless	0.5 [25]	0.5 [25]	–
$a_{s}$	m^2^ m^−3^	$2\varepsilon_{s}/R_{s}$	$2\varepsilon_{s}/R_{s}$	–
brug	unitless	1.5 [25]	1.5 [25]	1.5 [25]
$c_{e,0}$	mol m^−3^	1000 ^m^	1000 ^m^	1000 ^m^
$c_{s,max}$	mol m^−3^	22,860 [23]	22,860 [23]	–
$D_{e}^{eff}$	m^2^ s^−1^	$7.5\times 10^{-11}$ [25]	$7.5\times 10^{-11}$ [25]	$7.5\times 10^{-11}$ [25]
$D_{s}$	m^2^ s^−1^	$4\times 10^{-18}$ ^a^	$4\times 10^{-18}$ ^a^	–
$\varepsilon_{e}$	unitless	0.444 [25]	0.444 [25]	0.724 [25]
$\varepsilon_{s}$	unitless	0.36 ^m^	0.395 ^m^	–
F	C mol^−1^	96,485	96,485	96,485
k	mol^−1/2^ m^5/2^ s^−1^	$2.20728\times 10^{-5}$ [25]	$2.20728\times 10^{-5}$ [25]	–
$L_{s}$	m	$20\times 10^{-6}$ ^m^	$20\times 10^{-6}$ ^m^	−
$L_{SEP}$	m	−	−	${25\times 10^{-6}}^{m}$
N	unitless	4 ^a^	4 ^a^	–
R	J mol^−1^ K^−1^	8.3145	8.3145	8.3145
$R_{COAT}$	Ω m^2^	8.3 ^a^	0.8 ^a^	–
$R_{s}$	m	$8.5\times 10^{-8}$ ^m^	$8.5\times 10^{-8}$ ^m^	–
$S_{A}$	unitless	0.5 ^a^	1 ^a^	–
$\sigma^{eff}$	S m^−1^	3.8 [25]	3.8 [25]	–
$t_{+}^{0}$	unitless	0.363 [25]	0.363 [25]	0.363 [25]

m—measured or estimated parameters, a—approximated by the model, [link]—reference to the source of the parameters that are available in the literature.

**Figure A1:**
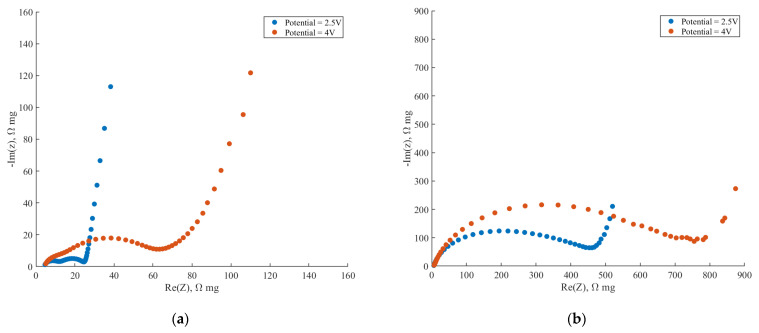
Electrochemical Impedance Spectroscopy (EIS) of (**a**) LFP electrode with a conductive binder and (**b**) conventional LFP electrode.

**Figure A2:**
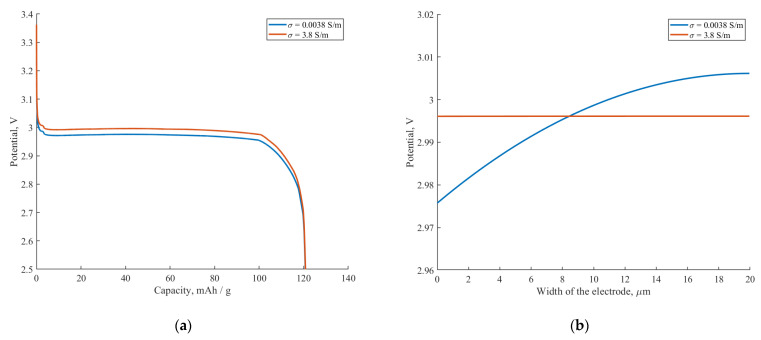
(**a**) The influence of the effective solid conductivity on the galvanostatic discharge curve (0.5C) of the simulated LFP material. (**b**) Potential distribution in the electrode material (at time = 2000 s from the beginning of discharge) for two different values of effective solid conductivity at 0.5C current.

## Data Availability

The data presented in this study are available on request from the corresponding author.

## Abbreviations

**Symbol**	**Interpretation**
A	geometric surface area of the electrode, [m^2^]
$A_{particle}$	particle surface area, [m^2^]
$a_{s}$	specific surface area of the porous electrode, [m^2^ m^−3^]
brug	Bruggeman coefficient, [unitless]
$c_{e,0}$	initial electrolyte salt concentration, [mol m^−3^]
$c_{s}$	concentration of lithium in the lithium iron-phosphate, [mol m^−3^]
$c_{s,0}$	initial concentration of lithium in the lithium iron-phosphate, [mol m^−3^]
$c_{s,99\%}$	concentration of lithium in the lithium iron-phosphate charged to 99%, [mol m^−3^]
$c_{s,max}$	maximum concentration of lithium in the iron-phosphate, [mol m^−3^]
$D_{e}$	diffusion coefficient of lithium ions in the electrolyte, [m^−2^ s^−1^]
$D_{e}^{eff}$	effective electrolyte diffusivity, [m^−2^ s^−1^]
$D_{s}$	diffusion coefficient of lithium in the lithium iron-phosphate, [m^−2^ s^−1^]
F	Faraday’s constant, 96485 [C mol^−1^]
$f_{A}$	activity coefficient of the electrolyte, [unitless]
j	lithium flux across interface, [mol m^−2^ s^−1^]
$i_{0}$	exchange current density, [A m^−2^]
I	applied current density, [A m^−2^]
L	cell width, [m]
$L_{s}$	width of the electrode, [m]
$L_{sep}$	width of the separator, [m]
$L_{Z}$	length of the lithium iron-phosphate cylindrical particle, [m]
N	number of ion-impermeable spots on the lithium iron-phosphate particle [unitless]
r	radial coordinate, [m]
R	universal gas constant, 8.31451 [J mol^−1^ K^−1^]
$R_{film}$	resistance of the film covering the active surface of the lithium iron phosphate, [Ω m^2^]
$R_{COAT}$	resistance of the coating of the lithium iron phosphate, [Ω m^2^]
$R_{s}$	radius of the iron-phosphate particle, [m]
$S_{A}$	active surface fraction of the lithium iron phosphate (allows ion intercalation) [unitless]
$S_{i}$	ion-impermeable surface fraction of the lithium iron phosphate [unitless]
t	time, [s]
$t_{90\%}$	diffusion time required to charge material up to 90% by potential step technique, [s]
$t_{+}^{0}$	transference number, [unitless]
T	cell temperature, [K]
$U_{OCP}$	open circuit potential, [V]
x	distance across the cell, [m]
z	distance along the cylindrical particle, [m]
α	Charge transfer coefficient, [unitless]
$\varepsilon_{e}$	volume fraction of the electrolyte in the electrode phase, [unitless]
$\varepsilon_{s}$	volume fraction of the LFP in the electrode phase, [unitless]
$\varepsilon_{sep}$	volume fraction of the separator in the separator phase, [unitless]
η	overpotential, [V]
θ	angular coordinate, [rad]
κ	electrolyte conductivity, [S m^−1^]
$\kappa^{eff}$	effective electrolyte conductivity, [S m^−1^]
σ	solid conductivity, [S m^−1^]
$\sigma^{eff}$	effective solid conductivity, [S m^−1^]
$\varphi_{e}$	potential in the electrolyte, [V]
$\varphi_{s}$	potential of the lithium iron phosphate, [V]

## Appendix A

This appendix details the equations used for the potential step simulation of a single-particle system. The radial diffusion equation has the following form:

(A1) $$D_{s}\cdot \nabla^{2}c_{s}\left(r,\theta ,t\right)=\frac{\partial c_{s}\left(r,\theta ,t\right)}{\partial t},$$

where $c_{s}$ is the solid concentration of Li^+^ ions, r is the distance from the center of the particle, t is time, and θ is the angular coordinate.

Initial condition for the Equation (A1) is as follows:

(A2) $$c_{s}\left(r,\theta ,0\right)=c_{s,0},$$

Boundary conditions corresponding to the Equation (A1) are as follows:

(A3) $$c_{s}\left(r=R_{s},\theta \in S_{A},t\right)=c_{s,99\%},$$

(A4) $$D_{s}\frac{\partial c_{s}(r=R_{s},\theta \notin S_{A},t)}{\partial r}=0,$$

The first boundary condition means that after applying the potential step, the concentration of Li^+^ ions reaches an equilibrium value for a given potential on the active surface of the LFP particle. The second boundary condition implies that the flux of Li^+^ ions is zero through the inactive surface of the particle.

## Appendix B

This appendix details the Electrochemical Impedance Spectroscopy (EIS) measurements of both samples. Figure A1 shows the Nyquist plots for the two LFP electrodes: the composite with a conductive binder (Figure A1a) and the conventional composite (Figure A1b). The composite with a conductive binder demonstrates high- and mid-frequency semicircles, which can be ascribed to charge transfer resistance $R_{CT}$ and coating resistance $R_{COAT}$, respectively. However, the conventional composite shows only a mid-frequency semicircle. This is, probably, because $R_{COAT}$ is much larger than $R_{CT}$ for the conventional composite; thus, the $R_{CT}$ is hardly distinguishable. One can see that the conventional composite electrode has at least 10 times higher resistance than the electrode with the conductive binder. Thus, the addition of a conductive binder and a reduction in the carbon and PVDF additives indeed facilitate the ion intercalation, which agrees with the results obtained in Section 3 of our work.

## Appendix C

This appendix details the impact of the effective solid conductivity on the voltage of the discharging cell. The value of the effective solid conductivity ($\sigma^{eff}$) of different LFP materials that can be found in the literature varies in a wide range (between 5 × 10^−4^ and 3.8 S/m [23,25,32]). Figure A2a compares two galvanostatic discharge curves of the LFP material calculated for the lowest and highest value of $\sigma^{eff}$ taken from the literature. The values of other parameters correspond to the positive electrode with a conductive binder (see Table 1). As one can see from Figure A2a, the lowest value of $\sigma^{eff}$ results in only a 30 mV shift in the discharge plateau. Higher values of effective solid conductivity result in an even lower drop in the discharge voltage. Thus, provided the electrode is relatively thin (2 × 10^−5^ m) and the active material is pre-coated, the effective solid conductivity does not have a significant effect on the cell performance. Figure A2b compares the potential distribution within the composite electrode for two values of $\sigma^{eff}$. As one can see, the lowest values of $\sigma^{eff}$ for the pre-coated material found in the literature give only a 30 mV change in the total potential of the cell.
